# What Difference Does Language Make? Comparing Systematic Evidence Reviews of Vietnamese and English Language Literatures on Climate Change and the Health of Outdoor Workers

**DOI:** 10.1111/area.70080

**Published:** 2025-12-16

**Authors:** Anh Ngoc Vu, Jonathan Rigg, Felicity Kersting, Ekaterina Tarnovskaya, Loc Duc Nguyen

**Affiliations:** 1 National Centre for Social Research, London, UK; 2 Human Geography, University of Bristol, Bristol, UK; 3 Social Life Research Institute, Ho Chi Minh City, Vietnam

**Keywords:** climate change, epistemic justice, health, outdoor workers, systematic literature reviews, Vietnam

## Abstract

The dominance of English-language scholarship in global research has led to a significant knowledge divide, often marginalising research from non-English-speaking regions, particularly in the Global South. This paper presents a comparative analysis of two systematic literature reviews (SLRs) on the health impacts of climate change on outdoor workers in urban Asia, focusing on English- and Vietnamese-language literature. By comparing these two bodies of scholarship, the study uncovers key thematic convergences and divergences, reflecting broader disparities in knowledge production, research norms and priorities. The English-language literature emphasises macro-level analyses, theoretical frameworks, and long-term policy interventions, while the Vietnamese-language literature offers more localised, context-specific insights, highlighting immediate coping strategies and health risks faced by outdoor workers. We argue that the systematic exclusion of non-English sources risks reinforcing existing epistemic hierarchies, narrowing debate on global health and climate change research. By integrating Southern-language perspectives, research can better and more equitably address the diverse vulnerabilities of outdoor workers and develop more effective and locally attuned interventions. The study emphasises the need to cast our linguistic net more widely to enrich global understanding and help achieve epistemic justice.

## 1 Introduction

The global intellectual landscape of academic research remains predominantly shaped by English-language scholarship, often marginalising contributions from non-English speaking regions, particularly those from the Global South (Connell 2007; de Sousa Santos 2015). This linguistic hegemony constrains the breadth of knowledge production and dissemination, limiting our capacity to engage fully with diverse perspectives and creating the risk of bias. Scholarship produced in Southern languages is frequently overlooked, which in turn inhibits the development of theoretical frameworks that account for the socio-economic, cultural, and environmental specificities of these regions. This marginalisation is particularly problematic in fields such as climate change, where vulnerable populations are predominantly in the Global South.

The absence of Southern academic voices and perspectives is multifaceted and well-documented (Smith 2021; Patel 2015). One area, however, where attention to date has been limited is with regard to systematic literature or systematic evidence reviews. The exclusion of non-European language literature from such systematic reviews is yet another means by which scholarship has narrowed its perspective. Jackson and Kuriyama’s (2019) assessment of 250 systematic reviews on PubMed revealed that 34% explicitly excluded non-English articles, 32% did not mention language exclusion but failed to include non-English language studies, while 34% claimed no language restriction. Of this latter 34% with no language restriction, however, only 22% actually incorporated non-English language articles, representing a mere 2% of the total studies surveyed. Implicit in this tendency to focus entirely or mostly on English, or at best European language sources, is an assumption that non-European literature will echo European language sources. This, in itself, is an assumption requiring further investigation. Furthermore, not engaging with research published in non-European languages risks reinforcing the hierarchical structure of knowledge production where insights from the South are framed merely as ‘local’ knowledge (de Sousa Santos 2015; Connell 2007), rather than as potentially contributory and equally valuable to global academic discourse.

This paper examines whether surveying a non-English language literature, namely Vietnamese, produces findings different from those in English. We do this through two systematic literature reviews (SLRs) on the health impacts of climate change on out-door workers in urban Asia. These reviews compare English- and Vietnamese-language literatures to examine the extent of convergence and divergence in the themes addressed, their relative balance or importance, and the interpretations that are drawn. As we will show, there are notable differences—as well as similarities—between the bodies of literature, challenging the assumption that beneath language and cultural divides, the science remains the same. This has implications for climate- and health-related policy interventions and, potentially, more widely, for theorisation.

The question that lies at the heart of the paper is deceptively simple: what similarities and differences are revealed in systematic literature reviews of sources in different languages? We focus our attention on how differences between the two SLRs illuminate different thematic priorities and knowledge production processes. We reflect on why these differences exist and their broader implications for conceptualising climate change–health impacts and policy interventions. At stake are not merely linguistic differences, but distinct epistemological orientations, methodological preferences, and assumptions about whose knowledge counts.

## 2 Methodological Notes

The reviews were conducted in connection with our Wellcome Trust-funded research project (227993/Z/23/Z) examining the impacts of climate change on the health of outdoor workers in four major cities in Vietnam. We undertook two parallel systematic literature reviews, one focusing on the English-language literature and the other on Vietnamese-language sources—using the same protocols. Due to limitations in Vietnamese databases, some adaptations were necessary, particularly in terms of keyword restrictions and lack of full-text search capabilities (see below). Despite these challenges, the overall methodology and inclusion criteria were consistently applied. Both reviews were designed to capture the breadth and depth of research on this topic, emphasising the intersection of climate change and health risks facing outdoor labourers in urban environments.

The first step involved a screening process, applying well-defined inclusion criteria to ensure the relevance of the selected studies. The criteria included employment type, specifically outdoor workers; geographical context, concentrating on urban regions in Asia; health impacts, such as heat stress, respiratory conditions, and other climate-related health risks; and types of literature, covering both relevant academic articles and grey literature. The evidence reviews covered the period from January 2000 to May 2024. Each identified study was evaluated for its applicability to maintain a consistent focus across the two reviews.

For the English-language review, the search parameters (Table 1) initially resulted in the identification of 734 papers across three databases: 417 papers in Scopus, 222 papers in Web of Science, and 95 papers in PubMed. After removing duplicates, 479 papers were screened at the title and abstract level and 26 papers were identified for full-text review. The full-text review selected 18 papers for subsequent extraction and analysis. The Vietnamese-language review (Table 2) found 88 papers in the databases, including 67 papers in Google Scholar, 11 papers in HCM VNU Lib (the EBSCO Discovery Service of the Central Library of Vietnam, National University Ho Chi Minh City) and 10 papers in MOST Pub Database (the National Database on Science and Technology of the Ministry of Science and Technology of Vietnam). Removing duplicates and screening at the title and abstract level excluded 42 papers, leaving 46 papers for the full-text review, with 8 identified as relevant for extraction and analysis.

The findings from both the English-language and Vietnamese-language literatures were synthesised to facilitate a comparative analysis. This involved identifying key themes, methodological approaches, and outcomes. Particular attention was given to differences in the geographical scope of the research, the specificity of health outcomes, and the contextual factors influencing the vulnerability of outdoor workers. While the English-language review drew on internationally recognised databases, the Vietnamese-language review focused on Vietnamese sources and databases, namely the HCM VNU Lib, the MOST Pub Database and Google Scholar.

One of the main challenges we faced was the keyword limits imposed by Vietnamese scientific databases. Google Scholar allows a maximum of 16 keywords per search, HCM VNU Lib limits searches to 12 keywords, and the MOST Pub Database to 8 keywords. Additionally, both HCM VNU Lib and the MOST Pub Database do not support full-text searches. To address these constraints, we refined our search strings, focusing on more general terms related to our research topics—climate, health, employment, society, and urban areas—resulting in a total of 15 keywords (see Table 2). For the HCM VNU Lib and MOST Pub Database, we split the searches into smaller groups to accommodate their keyword restrictions, then manually filtered the results to isolate studies specifically focused on urban areas in Asia.

## 3 Juxtaposing Vietnamese and English Scholarships

Both reviews examine outdoor workers in urban settings across Asia, with Vietnamese-language studies predominantly focused on formal-sector workers such as waste collectors, road traffic police, and construction workers. In contrast, the English-language studies include a broader range of worker types, including informal workers such as street vendors and home-based workers (Ko et al. 2020). In both literatures, how-ever, informal and precarious workers received limited focused attention.

It is important to note a distinction in the geographic scope of the two literatures. The Vietnamese-language studies were overwhelmingly concerned with Vietnam’s urban context. By contrast, the English-language literature was broader in its geographical reach, reaching across Asia and often aiming for cross-national or comparative generalisation. Nevertheless, both literatures converged thematically on the intersecting challenges of climate or weather variability, occupational health risks, and the vulnerabilities faced by outdoor workers in urban settings (Table 3). The search string for urban included ‘city’, ‘town’, ‘metropolitan’ and/or ‘urban’, although the papers themselves largely focused on larger urban centres (i.e., cities).

### 3.1 Points of Similarity

The English-language and Vietnamese-language reviews reveal several similarities in methodological approaches and findings. Both sets of studies rely on quantitative or mixed-methods methodologies; purely qualitative studies are scarce. The English-language review consisted of nine quantitative, eight mixed-methods and one qualitative study. In the Vietnamese review, there were six quantitative and two mixed-method studies; none took a purely qualitative approach. Second, a similar set of coping strategies was employed by outdoor workers to mitigate the effects of extreme heat, such as using protective clothing and equipment (Khan et al. 2023; Knowlton et al. 2014; Ha 2021; Hanh 2021). Air pollution was the second most common climate-related hazard explored in 17% of papers from each of the two reviews. These papers examined the respiratory impacts of air pollution, including bronchial disorders, chronic obstructive pulmonary disease, asthma, emphysema, lung cancer, and tuberculosis (Cheng and Huang 2019; Odihi 2001; Khan et al. 2023; Ninh et al. 2024; Anh 2021).

Third, there were resonances in the knowledge gaps in the literature identified in the two reviews. The focus on the adverse impacts of high temperature on workers’ health meant the impacts of other extreme weather events, such as flooding, droughts, storms, and cyclones were underplayed (Trien 2014; Nga et al. 2016; Hanh 2021; Anh 2021; Chau et al. 2021). Both literatures also paid scant attention to the particular vulnerabilities and exposures of informal, precarious and lower-wage workers (Dutta et al. 2015; Ko et al. 2020; Sheng et al. 2018; Chau et al. 2021; Nga et al. 2016; Ha 2021; Hanh 2021; Anh 2021; Suong and Thien 2024; Ninh et al. 2024). Several papers explicitly called for policy interventions to address the risks faced by outdoor workers, including the adoption of occupational heat standards, improved workplace protections, and better integration of worker needs into climate adaptation planning (Dutta et al. 2015; Lohrey et al. 2021). Finally, interdisciplinary and longitudinal research was absent (Ko et al. 2020; Nga et al. 2016; Ha 2021; Hanh 2021; Anh 2021; Suong and Thien 2024; Ninh et al. 2024).

## 4 Points of Difference

Despite these similarities, several notable differences emerged. Methodologically, the Vietnamese-language literature not only contained fewer studies overall—as might be expected—but also exhibited an even stronger bias toward quantitative approaches. Only one study employed mixed methods, and qualitative research was almost entirely absent, suggesting limited engagement with interpretive or experiential dimensions of climate-health vulnerabilities.

While fewer papers were eligible for inclusion in the Vietnamese-language review, these papers covered a wider range of topics. The methodological approach was more narrowly drawn, but the concerns addressed were wider. Both reviews showed a focus on heat phenomena, which were examined by 61% of papers in the English-language review and 37% of papers from the Vietnamese-language study. Heat-related events and impacts including heat stress, heat stroke, and heat exhaustion dominated both SLRs, but even more so in the English-language studies (Dutta et al. 2015; Li et al. 2021; Lohrey et al. 2021; Sheng et al. 2018; Cheng and Huang 2019; Knowlton et al. 2014; Liu et al. 2019; Yin and Wang 2017; Yu et al. 2023; Trien 2014; Nga et al. 2016; Hanh 2021; Anh 2021). The remaining papers in the Vietnamese-language review included a broader swathe of weather phenomena such as extreme cold (Trien 2014; Ha 2021), air and noise pollution (Ninh et al. 2024; Anh 2021), and general bad weather (Suong and Thien 2024). ‘Weather’ was less meteorological in the Vietnamese literature. This may reflect a more nuanced view of the weather-related challenges faced by outdoor workers.

In a similar vein, the Vietnamese-language review identified a broader array of health impacts of climate change than its English-language counterpart. These included vestibular disorders (Chau et al. 2021; Ninh et al. 2024), digestive problems (Ninh et al. 2024), urinary diseases (Ha 2021; Anh 2021; Chau et al. 2021), musculoskeletal conditions (Ha 2021; Chau et al. 2021; Ninh et al. 2024), and dengue (Chau et al. 2021). In contrast, the English-language literature tended to operationalise health impacts through more medicalised proxies such as hospital admissions (Cheng and Huang 2019) and mortality rates (Ko et al. 2020). We suggest that ‘health’ in the Vietnamese context is conceptualised more expansively and less through a strictly biomedical lens, allowing for a wider inclusion of symptoms, illnesses, and locally perceived risks. This broader framing extended the scope of what is counted as a climate-related health impact and speaks to Vietnam’s more holistic medical tradition, in which concepts from Traditional Chinese Medicine (TCM) remain influential (Monnais-Rousselot et al. 2012). However, this inclusivity does not extend to how vulnerability is theorised. In this regard, the English-language literature offers more complex and intersectional analyses. Studies frequently examine how gender, age, migration status, and precarious employment interact to shape differential exposure and capacity for adaptation (Cheng and Huang 2019; Lohrey et al. 2021), with particular emphasis on the heightened vulnerabilities faced by women and migrant workers. The Vietnamese-language literature, by contrast, offers limited engagement with such intersectional dimensions. While some studies report basic demographic variables such as age or years of service (e.g., Ha 2021; Anh 2021), few analyse how these characteristics intersect or contribute to differentiated vulnerability. Notably, there is minimal discussion of gendered labour, migration status, or disability. This reflects a broader trend: a predominant focus on formal-sector workers, with only one study explicitly addressing informal labour. Given that 68.5 per cent of Vietnam’s workforce is informally employed amounting to approximately 33.6 million people (ILO 2023) this represents a significant analytical omission.

The English-language studies had a more resolute focus on macro-level interventions such as community support and education (Ko et al. 2020; Wu et al. 2022), or interventions such as altering work hours, mandated through policy (Knowlton et al. 2014). The studies identified in the Vietnamese-language review paid greater heed to individualised strategies such as exercising regularly, taking micronutrients, maintaining posture, and frequently changing positions at work (Ha 2021). These differences hint at matters of responsibility and accountability: to whom does the responsibility of addressing such health impacts, fall?

As with coping strategies, the focus for policy recommendations in the English- and Vietnamese-language reviews diverged. Both recommended the promotion of information and additional education about the impacts of climate change on health (Hanh 2021; Ha 2021; Paul et al. 2019; Khan et al. 2023; Knowlton et al. 2014; Odihi 2001; Lohrey et al. 2021). Beyond this, the English-language recommendations mainly focused on city-wide, national, or regional interventions such as improved data infrastructure and monitoring (Khan et al. 2023; Cheng and Huang 2019; Dutta et al. 2015); setting heat exposure limits internationally and regionally (Dutta et al. 2015; Yin and Wang 2017; Cheng and Huang 2019); and urban planning to address the needs of vulnerable groups (Paul et al. 2019).

Recommendations from the Vietnamese-language review focused more on the conditions of workers, including the need to improve working environments (Nga et al. 2016; Suong and Thien 2024); the provision of additional protective equipment (Anh 2021); ensuring working shifts are appropriate lengths (ibid.); and utilising technological advancements to reduce outdoor work exposure (Ha 2021; Anh 2021). The Vietnamese-language papers paid attention rather more to short- and medium-term behavioural adjustments, often enacted in places of work; the English-language literature placed a greater onus on longer-term structural changes.

Finally, and this echoes a point made earlier, the Vietnamese literature addressed the Vietnam context, with rarely any attempt to compare and contrast Vietnam to other countries’ experiences. This inward orientation constructs Vietnam as a distinctive case without positioning it within a broader regional or global frame. In the English language literature, the tendency is reversed: arguments for exceptionalism are avoided in the interests of comparison.

## 5 Discussions

This comparative analysis of the English- and Vietnamese-language SLRs cautions against the assumption that research conducted in one language can serve as a mirror for knowledge produced in other linguistic registers. Literature on the health impacts of climate change on outdoor workers diverged in important ways, and this reflects epistemological, cultural, and political differences.

The Vietnamese-language studies tend to be more descriptive, focused on formal sector workers, and framed around individual behaviours. In contrast, English-language studies more often adopt structural and intersectional approaches, high-lighting systemic inequalities and the role of institutions. These differences stem in part from linguistic and cultural norms. For example, Vietnamese academic writing favours less direct argumentation than English. Political context is also important in a national context where self-censorship is common and overt criticism of government or policy is avoided. Language shapes how knowledge is communicated. Politics dictates what can be said.

Existing literature on the knowledge divide between the Global North and Global South provides potential explanations for the differences observed in the reviews. One key distinction lies in climate research and policy priorities: while the Global North tends to focus on the long-term risks and systemic consequences of climate change, research in the Global South is more concerned with short-and medium-term adaptation strategies (Skelton et al. 2018; Pasgaard et al. 2015). This likely reflects the more immediate, tangible impacts of climate variability on health, shelter, and livelihoods in many Southern contexts, as opposed to the often more abstract or projected future risks emphasised in Northern literature (Redclift and Sage 1998). This contrast is evident in our reviews. For instance, English-language papers frequently advocate for large-scale, structural reforms—such as urban planning for climate-resilient infrastructure (Paul et al. 2019) or national-level heat exposure standards (Cheng and Huang 2019; Dutta et al. 2015). By contrast, Vietnamese-language papers are primarily concerned with context-specific, occupational adaptations that can be implemented in the short term—for example, improving protective gear, adjusting work hours, or modifying individual behaviour to mitigate immediate health risks (Ha 2021; Anh 2021; Suong and Thien 2024). These distinctions reflect how climate change is not only experienced differently across contexts but also studied and framed according to varying institutional, political, and epistemic priorities.

The respective audiences of the English- and Vietnamese-language reviews also have an influence in shaping the two literatures. International publishing norms emphasise global perspectives, cross-country comparison and theoretical debates. This requires authors to take their case study material into the global arena, privileging generalisation over specification. Vietnamese-language outlets assume a geographically much more limited audience, one attuned to and concerned with the particularities of the Vietnamese case and the need to address Vietnamese policy challenges. This might explain, for instance, the willingness of the authors of the Vietnamese-language sources to cast their meteorological and health nets more widely than the international literature. Matters of definition and reach shape both literatures. So, it is not just that the Vietnamese-language literature is ‘closer’ to the ground, and therefore more granular. The English-language literature conformed to international conventions regarding where knowledge boundaries are drawn, in a manner that was not the case in the Vietnamese-language literature. As we note, for example, health impacts and interventions in the Vietnamese-language literature are informed by the norms of ‘Vietnamese’ medicine, without the need for reductionism to the accepted norms of Western (i.e., global) medicine. In effect, the literatures sit in different knowledge silos, ring-fenced by language.

These divergences extend to methodological choices, particularly the striking absence of qualitative research in the Vietnamese-language literature. This appears paradoxical: although these studies often provide detailed accounts of specific occupational settings and coping strategies, they rely almost exclusively on quantitative methods. This raises a broader question about the nature of ‘local detail’ and the types of evidence used to produce it. While descriptive and context-specific, the Vietnamese studies tend to document *what* workers do, but rarely interrogate *why* they do so, or *how* these responses are shaped by social position, affective experience, or structural constraint. Notwithstanding Vietnamese-language authors’ (and audiences’) close experiential proximity to the research subjects and local conditions, the reliance on quantitative data limits the ability of such research to explore the deeper processes through which climate impacts are understood, embodied, and navigated by workers and the subjective dimensions of climate-health linkages. The English-language studies, by contrast, show a greater openness to qualitative and mixed-methods approaches, allowing for a more sustained engagement with such interpretive dimensions.

The English-language literature prioritises macro-level analyses and theoretical paradigms with a predisposition toward generalisation and abstraction, wherein the focus gravitates toward universal principles and large-scale policy interventions. In the process there occurs a weeding out of matters that might be regarded as culturally distinct or geographically specific—’parochial’. The emphasis in the Vietnamese-language literature, by contrast, was on a nuanced understanding of climate-related challenges, context-specific issues, the particularities of the daily lives of outdoor workers and their resourcefulness in terms of diet and exercise, and practical local interventions that might not scale up.

## 6 Conclusion

The systematic exclusion of Southern-language literatures from international debates and theory-building in fields such as climate change and health has implications for both academic inquiry and policy-making. This paper highlights how Southern scholarship, exemplified by papers identified in our Vietnamese-language SLR, not only complements but also provides an alternative perspective. Perhaps the most straight-forward lesson is that ‘systematic’ literature (or evidence) reviews that exclude non-European (or non-English) language sources are systematically limiting. The ability to search databases of hundreds of thousands of sources, which can then be whittled down using carefully modulated search and inclusion/exclusion criteria, gives the impression of inclusiveness. We are then reassured that the arbitrary choices and preferences of traditional literature reviews have been avoided. This paper and the two systematic reviews that underpin it caution against such a view.

The reasons for the differences that have been revealed are multiple. They reflect conventions in the mainstream (English language) academic publishing world that create a necessity to say something ‘more’ and ‘wider’, with Southern voices and conditions being ironed out. More importantly, we note an insidious—subtle, hidden but potentially harmful—way that systematic literature reviews under the pretext of inclusive objectivity may actually reinforce certain views and perspectives, and marginalise others. The SLR methodology is deceptively reassuring: ‘The SLR utilises extracting search strategies to *make certain* that the *maximum extent* of relevant research has been considered’ in which ‘original articles are methodologically appraised and synthesised using *strict methods to locate, appraise and synthesise all research on a topic*’ (O’Brien and Mc Guckin (2016), [emphases added]). Such statements reassure the reader and researcher that every stone has been turned over.

Including Southern-language literature in systematic reviews is not a simple matter, but if our conclusions resonate more widely, then it is important for effective interdisciplinary and cross-cultural research and climate interventions. Such literatures are often not held by the main academic databases. Their interpretation requires language expertise and a good deal of work across the translation divide, which we have not considered. Nor, of course, are such literatures necessarily more attuned to the needs of those we seek to understand. Indeed, we see in the Vietnamese literature a tendency to overlook structural causes of vulnerability. Simply expanding the linguistic scope of systematic reviews is not, on its own, sufficient to address the deeper partialities embedded in knowledge production—and this concern extends to global synthesis efforts such as those led by the IPCC. While recent reports have made efforts to diversify authorship and broaden the evidence base, the dominance of English-language, peer-reviewed literature continues to shape what is included in assessments and what is left out. This creates a persistent bias toward countries, institutions, and disciplines with the resources and infrastructure to produce internationally legible knowledge. As a result, locally grounded insights—often published in non-English languages and embedded in context-specific epistemologies—remain peripheral to global debates, even as they are critical for effective adaptation planning in the Global South. Moreover, prevailing modes of synthesis frequently reproduce broader tendencies within climate science: limited interdisciplinarity, superficial engagement with participatory or co-produced approaches, and a persistent emphasis on economic metrics. As Rigg and Mason (2018, 1031) argue, confronting such reductionisms opens the possibility for ‘a different kind of climate science—one characterized by deep interdisciplinarity, meaningful public engagement and the recognition that global processes have distinct local signatures’. Attending more carefully to the politics of language in systematic reviews is one small but necessary step toward that broader reorientation.

For geographical research in particular, the findings highlight the need to engage more reflexively with the politics of method, voice, and translation. Geography’s strength lies in its attention to scale, place, and power—yet SERs risk flattening difference in the pursuit of comparability. A more pluralistic approach to synthesis would attend not only to linguistic diversity but also to epistemological multiplicity, embracing research that is situated, embodied, and often resistant to quantification. Recognising language as a structuring force in knowledge production is a first step. But the broader challenge is to develop review practices—and disciplinary norms—that are meaningfully inclusive of the multiple ways in which climate-health relations are lived, known, and theorised.

## Figures and Tables

**Table 1:** Search strings for English-language review.

Component	Search strings
Climate	Climat* OR weather OR flood* OR drought OR heatwave OR heat OR cold OR global warming OR hurricane OR typhoon OR monsoon OR rainfall OR adaptation OR mitigation OR greenhouse gas OR storm OR cyclone OR coastal erosion OR risk OR natural disaster OR wildfire
Health	Health OR disease OR infectious OR morbidity OR wellbeing OR quality of life OR mortality OR death OR accident OR well-being OR injur* OR hospital* OR mental OR medical* OR emergency OR stroke OR maternal OR exhaustion OR cramp OR pregnan* OR fever* OR fatigue OR burn OR stress OR skin
Work	Outdoor work* OR porter OR construction OR vendor OR street vendor OR rider OR outdoor occupation
Social	Gender OR age OR generation OR old OR young OR elderly OR poverty OR exclusion OR precari* OR migrant OR minority OR vulnerabl* OR informal* OR casual* OR contract* agreement
Urban	City OR cities OR town OR urban OR metropolitan
Asia	Asia OR Vietnam OR China OR Timor Leste OR East Timor OR Indonesia OR Philippines OR Cambodia OR Thailand OR Laos OR Lao PDR OR Myanmar OR Burma OR Malaysia OR Brunei OR Brunei Darussalam OR Mongolia OR India OR Sri Lanka OR Nepal OR Pakistan OR Bangladesh OR Bhutan OR Afghanistan OR North Korea OR Democratic People's Republic of Korea

**Table 2:** Search strings for Vietnamese-language review.

Filter		Main keywords		Vietnamese synonyms and relevant keywords
		English	Vietnamese	
All main keywords were combined for searching in one string with synonyms and relevant keywords included.		Climate	khí hậu	“khí hậu” OR “thời tiết” OR “thiên tai”
	AND	Health	sức khỏe	“sức khỏe” OR “bênh” OR “triêu chứng”
	AND	Work	viêc làm	“làm viêc ngoài trời” OR “lao động ngoài trời” OR “viêc làm ngoài trời”
	AND	Society	xã hội	“xã hội” OR “con người” OR “yếu thế”
	AND	Urban	đô thị	“đô thị” OR “thành phố” OR “thị xã”
Each word in this group was manually added along with the string of keywords above for searching.	AND	Asia	Châu Á	”Châu Á“ OR” Viêt Nam” OR” Trung Quốc” OR” Timor Leste” OR” đông Timor” OR” Indonesia OR” Philippines” OR” Campuchia” OR” Thái Lan” OR” Lào” OR” CHDCND Lào” OR” Myanmar” OR” Miến điên” OR” Malaysia” OR” Brunei” OR” Brunei Darussalam” OR” Ấn độ” OR” Sri Lanka” OR” Nepal” OR” Pakistan” OR” Bangladesh” OR” Bhutan” OR” Afghanistan” OR” Bắc Triều Tiên” OR” Cộng hòa Dân chủ Nhân dân Triều Tiên” OR” Mông Cổ”

**Table 3:** Comparative synthesis.

	English-language literature	Vietnamese-language literature	Notes
Methods	Quantitative and mixed-methods; some qualitative	Exclusively quantitative; no qualitative studies	Vietnamese-language literature prioritises prediction over interpretation; qualitative insights largely absent
Weather framing	Focused on heat-related impacts	Broader framing of what counts as ‘climate’	Vietnamese literature is less meteorological in focus and more context-sensitive (cultural and linguistic factors at work)
Health outcomes	Uses mortality and hospitalisation as proxies for health impact	Identifies specific ill-health conditions (urinary, musculoskeletal, digestive, vestibular) and broader in scope	Vietnamese literature is less medicalised and defines ill-health more broadly and practically
Worker groups studied	Includes informal workers (e.g., street vendors, home-based garment workers)	Primarily formal sector (e.g., sanitation, police, construction)	Informal and precarious workers are underrepresented in Vietnamese-language studies
Causes of vulnerability	Intersectionality (gender, age, income, migrant status); emphasis on social structures	Focus on environmental exposure and formal working conditions	Different understandings of who is vulnerable and why; less attention to structural inequality in Vietnamese literature
Coping strategies and preferred responses	Institutional: policy-led, early warning systems, community-level adaptation	Individualised: PPE, micronutrients, posture changes, rest	Vietnamese-language studies focus on self-directed behavioural adaptation; English-language studies highlight system-level interventions
Adaptation responsibility	Onus on government, employers, and society	Onus on the individual (e.g., exercising regularly, taking micronutrients, maintaining posture, changing positions at work)	English-language literature emphasises shared and systemic responsibility; Vietnamese-language literature assumes individual agency
Policy orientation	Emphasis on scalable, long-term policies (urban planning, standards)	Focus on workplace and short- and medium-term behavioural adjustments	English-language sources emphasise the generalisable lessons and longer term; Vietnamese-language sources stay close to context-specific, practical fixes and the shorter term

## Data Availability

No new data were created or analysed in this study. All data sources reviewed are publicly available through academic databases and are fully cited within the manuscript and Supporting Information.
